# Exclusive Breastfeeding and Vitamin D Supplementation: A Positive Synergistic Effect on Prevention of Childhood Infections?

**DOI:** 10.3390/ijerph19052973

**Published:** 2022-03-03

**Authors:** Raffaele Domenici, Francesco Vierucci

**Affiliations:** Pediatric Unit, Saint Luca Hospital, 55100 Lucca, Italy; raf.domenici@gmail.com

**Keywords:** breastfeeding, human milk, infections, vitamin D, supplementation, COVID-19

## Abstract

Human milk is the best food for infants. Breastfeeding has been associated with a reduced risk of viral and bacterial infections. Breast milk contains the perfect amount of nutrients needed to promote infant growth, except for vitamin D. Vitamin D is crucial for calcium metabolism and bone health, and it also has extra-skeletal actions, involving innate and adaptive immunity. As exclusive breastfeeding is a risk factor for vitamin D deficiency, infants should be supplemented with vitamin D at least during the first year. The promotion of breastfeeding and vitamin D supplementation represents an important objective of public health.

## 1. Introduction

Human milk has an undisputed biological and nutritional primacy: it is the first food consumed by infants; it is safe, sustainable, and fair. The World Health Organization (WHO) identified the improvement in the quality of life of mothers and children as one of the world’s primary health objectives and indicated that the promotion of breastfeeding is a fundamental choice for health. Indeed, increasing the breastfeeding rate, in addition to showing the highest cost–benefit ratio, is one of the most important interventions in terms of efficacy [1,2]. Promoting breastfeeding is the single most effective intervention in preventing infant deaths: it has been estimated that breastfeeding implementation could save 820,000 children under 5 years of age worldwide (87% of them younger than 6 months) and would reduce infection-related mortality by 88%, mostly due to reducing cases of respiratory tract infections and diarrhea, in infants under 3 months of age [3]. Human milk is a species-specific food, balanced in nutritional components, microbiologically safe, immediately available, and cheap. Moreover, it has epigenetic actions significantly contributing to the prevention of many diseases that may affect children, not only in early childhood but also later in life. Breastfeeding is associated with a reduced risk in both viral and bacterial infections, sudden infant death syndrome, obesity, diabetes, allergies, and cancer [1,4]. The benefits for mothers are also important, helping to reduce the risk of breast and uterine cancer and type 2 diabetes, and mitigating the vascular risk related to pregnancy [4,5]. Conversely, lactation failure is associated with a significant increase in infant morbidity and related costs, estimated to be one billion US dollars per day [6]. The ecological benefits of breastfeeding, secondary to the reduced consumption of environmental and energy resources for the production, packaging, transport, and distribution of formula milk, and the consequent waste disposal, are also significant [7]. Therefore, a call to action is advisable for governments, international organizations, and civil society to increase funding to support breastfeeding promotion, dissemination, and support.

Human milk contains the appropriate amounts of several nutrients needed to promote infant growth, except for vitamin D which plays a fundamental role in regulating phospho-calcium homeostasis, bone mineralization, and bone mass acquisition [8]. Beyond these classic “skeletal” functions, vitamin D also exerts various “extra-skeletal” actions. Indeed, vitamin D contributes to the expression of more than 1000 genes involved in regulation of immune response, cell growth and differentiation, and metabolism [9,10]. Among these “new” actions of vitamin D, the regulation of innate and adaptive immunity plays a leading role during pediatric age, as an adequate vitamin D status was found to be protective against both bacterial and viral infections [10,11,12]. Furthermore, the role of vitamin D in regulating immunity has been highlighted by association studies that correlated vitamin D deficiency with the risk of developing autoimmune diseases such as type 1 diabetes mellitus, multiple sclerosis, Chron’s disease, and rheumatoid arthritis [9,10,12,13]. In this review we discuss the role of breastfeeding and vitamin D supplementation in reducing the risk of infections in childhood.

## 2. Search Strategy

A literature search was performed using the electronic database of MEDLINE in December 2021; this was restricted to articles published in English. Publications before January 2010 were not considered to exclude older references possibly not reflecting current knowledge/evidence. Search terms focused on words related to breastfeeding, vitamin D, and pediatric common infections. Particularly, to identify original studies assessing the relationship between breastfeeding and childhood infections, we used “breastfeeding”, “human milk”, “newborns”, “infants”, “children”, “fever”, “mortality”, ”infections”, “respiratory infections”, “acute otitis”, “pneumonia”, “bronchiolitis”, “gastrointestinal infections”, “diarrhea”, “immunodeficiency virus”, “SARS-CoV-2”, and “COVID-19” as keywords. Systematic reviews and meta-analyses were also selected. To discuss vitamin D role in preventing infectious diseases in pediatric age we added the terms “vitamin D”, “cholecalciferol”, and “supplementation” to the keywords previously listed. For vitamin D our search was limited to meta-analyses, due to the considerable number of published studies.

## 3. Breastfeeding and Infections

Breast milk has been described as a complex and highly variable bioactive fluid, with changes in composition depending on the stage of lactation (from colostrum to late lactation), time of day, and maternal nutritional status. Moreover, breast milk is a source of bioactive molecules, bacteria, and immune cells (including macrophages, T cells, stem cells, and lymphocytes) that enhance immune maturation and protect the newborn against infections and inflammation [14]. Indeed, in addition to essential nutrients for early growth and development, human milk contains various immunologic components, such as α-lactalbumin, lactoferrin, lysozyme, and secretory immunoglobulin (Ig) A [15,16]. Colostrum, with its anti-inflammatory and anti-infectious properties, is particularly important during early postnatal life when neonatal adaptive immune system is still immature and ineffective to protect against pathogens [17].

Human milk shows high inter-individual variability, with the most profound changes observed in lipids composition, including long-chain polyunsaturated fatty acids which have showed immune-regulatory properties [18]. While several infant-related factors (i.e., birth weight, gestational age, and infant age) are known to affect nutritive and non-nutritive components of breast milk, there is limited or conflicting evidence regarding the possible role of maternal factors (i.e., maternal lifestyle, obstetric history, and medical conditions), except for mother’s diet that significantly influences milk composition. For example, at present there is inconsistent evidence that maternal atopy/allergy may affect breast milk composition of interleukins, growth factors, pro-inflammatory markers, cytokines, and fatty acids [19,20,21,22,23,24,25,26,27,28,29]. However, it has been reported that human milk growth factors and cytokines levels varied between populations for unknown reasons, and breast milk mediator levels declined at different rates postpartum, suggesting specific biological roles for human milk growth factors and cytokines in early postnatal development [30].

In Table 1, we summarized studies on breastfeeding and infections published since 1st January 2010 [31,32,33,34,35,36,37,38,39,40,41,42,43,44,45,46,47,48,49,50,51,52,53,54,55,56,57,58]. WHO recommended that all mothers should be supported to initiate breastfeeding as soon as possible after birth, within the first hour after delivery [59]. The early onset of breastfeeding is a simple, but effective, intervention to significantly improve neonatal morbidity/mortality outcomes, as reported by a systematic review of 18 studies. Particularly, breastfeeding was associated with lower risks of all-cause neonatal mortality (also among low-birth-weight babies), and infection-related neonatal mortality [60]. Breastfeeding help to restore intestinal microbiota in newborns from cesarean section, in which Bifidobacterium is less represented, with consequent reduction risk of contracting respiratory infections and diarrhea in early childhood [43]. The importance of promoting exclusive breastfeeding has been reinforced by a meta-analysis (13 studies; n = 46,499), finding that infection-related mortality risk in the first 5 months of life was higher in predominantly, partially, and non-breastfed infants compared to the exclusively breastfed ones. Moreover, non-breastfed children aged 6–23 months had higher risk of all-cause and infection-related mortality than children who continued breastfeeding [61]. The sudden protective effect of breastfeeding was reported by a retrospective case–control study, enrolling 140 infants aged <1 month. This study showed that exclusive or predominant breastfeeding, as opposed to formula or partial breastfeeding, significantly reduces the risk of neonatal fever-related hospitalization by over two-fold [32].

A combined action of peer-support groups and International Board-Certified Lactation Consultants is essential to promote breastfeeding support strategies, to enhance maternal empowerment, and to increase the knowledge of the protective effect of human milk against infections [62,63]. Particularly, peer-support for breastfeeding is associated with longer duration of exclusivity. Not surprisingly, breastfeeding promotion for low-birth weight babies in critical care is also cost-effective, being associated with lower costs and greater health benefits for mothers and infants [64]. Exclusive breastfeeding has been associated with risk reduction of gastrointestinal infections in late preterms [48]. The implementation of steps 1–9 of the Baby Friendly Hospital Initiative (BFHI) was associated with a significant reduction in frequency of mild and severe episodes of diarrhea and respiratory infections in infants younger than 6 months in Democratic Republic of Congo. Promoting BFHI steps 1–9 was also associated with a decreased incidence of both health facility and hospitalizations due to diarrhea and respiratory illness [42]. Recently, it has been estimated that failing to comply with WHO recommendations for breastfeeding entails a healthcare system cost of 118 million US dollars annually for the treatment of diarrhea and pneumonia/respiratory disease in Indonesia [65].

Despite early pacifier use being associated with breastfeeding discontinuation, no significant association was found with respiratory infections, even if constant pacifier use was shown to correlate with a slightly higher risk of coughing and wheezing [66].

Several studies evaluated the possible preventive action of natural breastfeeding against infectious diseases in early childhood, but only few extended follow-ups to second and third infancy [31,41,44,46,47,52,54,55,56]. Some studies suggested that the protective effect of human milk was maximum during the first 6–12 months of life (Table 1), while others found that significant reduction in infections risk lasted up to the age of 2 years or even beyond [31,41,44,55,56]. Conversely, in the cohort study of Tarrant, M. et al. (8327 children followed until 8 years of life) breastfeeding status at 3 months was not associated with hospitalization for infectious diseases beyond 6 months of age [46].

### 3.1. Breastfeeding and Respiratory Tract Infections

Several studies assessed the association between breastfeeding and the risk of upper and lower respiratory tract infections in childhood, most of which found a significant protective role of human milk, despite different applied methodology and various populations enrolled (Table 1). Predominant breastfeeding for 3–6 months was associated with a significant reduction in contracting respiratory infections during the first 6 months of life [45]. Similarly, a Spanish cohort study (580 children evaluated from birth to 14 months) confirmed that predominant breastfeeding for 4–6 months was associated with a lower risk of wheezing, low respiratory tract infections, and atopic eczema between 7 and 14 months of life [49].

A systematic review of 13 studies in Asian infants confirmed that breastfeeding compared to infant formula was associated with significantly lower rates of respiratory tract infections and diarrhea in the first year of life [67]. A recent nationally representative survey in Ethiopia (1034 infants aged < 6 months) demonstrated that exclusively breastfed subjects had a significant reduction in the frequency of illness with fever in the last 2 weeks compared to non-exclusively breastfed infants. Particularly, exclusively breastfed babies had lower odds ratio (OR) of having an illness with cough (OR 0.38) and diarrhea (OR 0.33) [57]. This finding agreed with the results of a large USA prospective longitudinal study (6861 children with a follow-up of 4 years) that found an inverse significant association between breastfeeding and the risk of respiratory infections with fever (OR 0.82), otitis media (OR 0.76), and infectious gastroenteritis (OR 0.55) at 3–6 months of life. Breastfeeding within any 3-month period was inversely associated with ear infection, gastroenteritis, conjunctivitis, laryngitis, and tracheitis also at 6–18 months. Finally, exclusive breastfeeding duration was weakly inversely associated with the risk of otitis media up to 48 months of age (OR 0.97). Taken together, these results suggest that breastfeeding can provide a mild protection against infections also after the first 6–12 months of life [47].

A Brazilian ecological study showed that prevalence of both exclusive breastfeeding among children under 6 months and breastfeeding among children 9–12 months-old were associated with a lower risk of hospitalization for pneumonia during the first year of life [33]. A meta-analysis confirmed that pneumonia mortality was higher among non-breastfed compared to exclusively breastfed infants aged < 5 months, and among non-breastfed compared to breastfed infants and young children aged 6–23 months [68]. These results reinforced the importance of promoting exclusive breastfeeding during the first 6 months of life and continuing breastfeeding thereafter.

A recent Indonesian retrospective case–control study found that 7–12 months-old non-breastfed infants had a 14 times higher risk of contracting respiratory infections [37]. Another prospective study evaluated 926 Greek children, recording feeding modalities and infectious episodes (acute respiratory tract infections, acute otitis media, gastroenteritis, urinary tract infections, conjunctivitis, candidiasis) during the first year of life. Children exclusively breastfed for 6 months had fewer infectious episodes (particularly respiratory infection and acute otitis media) and hospital admissions than those who were partially or non-breastfed. On the other hand, partial breastfeeding was not related to any protective effect against infections [58].

The French EDEN mother–child study, a cohort study with 8 years of follow-up, did not demonstrate a significant protective effect of breastfeeding on longitudinal patterns of cold/nasopharyngitis, skin rash, or respiratory symptoms. However, ever-breastfed infants had a significant lower risk of diarrhea in early infancy and bronchitis/bronchiolitis throughout infancy compared with never breastfed infants. Only predominant breastfeeding duration was related to frequent events of bronchitis/bronchiolitis and infrequent events of otitis [52].

In a USA prospective longitudinal study (1281 subjects followed until 6 years of life), children breastfed longer than 9 months had lower risk of past-year ear (OR 0.69), throat (OR 0.68), and sinus (OR 0.47) infections compared with those breastfed less than 3 months [56]. A meta-analysis confirmed that breastfeeding protects against acute otitis media until 2 years of life, and exclusive breastfeeding for the first 6 months was associated with higher risk reduction (43%) [69]. More recently, a Turkish cohort study (411 children evaluated up to 5 years of life) showed that breastfeeding longer than 12 months significantly reduced the risk of acute otitis media and acute gastroenteritis [55].

A large cohort study from UK (4040 children aged 1.00–1.99 years) evaluated the prevalence of frequent colds (>6 episodes), ear infections and croup within the last 12 months, and any episodes of bronchiolitis or pneumonia in relation with breastfeeding duration. This study found limited evidence of a protective effect of breastfeeding against all types of respiratory tract infections during the first 2 years of life, but results suggested that prolonged breastfeeding (>6 months) might protect against bronchiolitis (risk reduction of 28%) [38]. The reasons for this different efficacy of breastfeeding in reducing only bronchiolitis risk are not fully understood; however, another retrospective study (411 infants, age < 1 year) showed that the risk for requiring oxygen therapy to treat respiratory syncytial virus (RSV) bronchiolitis was significantly higher in the artificial-milk-formula-fed group than in the breastfed group [35]. A recent cohort study in Spain (969 infants) showed that any breastfeeding was significantly associated with a lower incidence of bronchiolitis and number of episodes of bronchiolitis in the first year of life, confirming that breastfeeding may represent an effective primary prevention strategy against bronchiolitis [36].

The protective role of breastfeeding against bronchiolitis is particularly relevant during actual coronavirus disease 2019 (COVID-19) pandemic. In 2020, a dramatic reduction in RSV bronchiolitis hospitalization was reported worldwide, coinciding with the spread of SARS-CoV-2 infection [70,71,72]. The most accredited hypothesis to explain this uncommon finding was that the strict adoption of non-pharmaceutical interventions to contain SARS-CoV-2 diffusion (including handwashing and social distancing) also reduced the circulation of other infectious agents, such as RSV [71]. Unfortunately, this reduction was only transient, with subsequent rebound during the fall and winter seasons in 2021–2022 [72,73,74]. For example, a recently published French study reported a delayed RSV epidemic in the period usually corresponding to the end of the epidemic season [75].

Breastfeeding and parent-reported hospitalizations, bronchiolitis and otitis events, and antibiotic use were prospectively collected up to 2 years among 9703 young children from the nationwide Etude Longitudinale Française depuis l’Enfance (ELFE) birth cohort. This study showed that the number of bronchiolitis events was not significantly related to ever breastfeeding or to breastfeeding duration, but predominant breastfeeding duration tended to be negatively related to the risk of frequent bronchiolitis events. Similarly, both any and predominant breastfeeding were not related to frequent otitis events. In contrast, any breastfeeding duration < 3 months was associated with higher risks of hospitalizations from gastrointestinal infections or fever, predominant breastfeeding duration < 1 month was associated with higher risk of a single hospital admission, and ever breastfeeding was associated with lower risk of antibiotic use, confirming a lower risk of infectious morbidity related to breastfeeding duration [53]. However, in another cross-sectional study ever breastfeeding compared with exclusive formula feeding was associated with decreased risk (−36%) of a lower versus upper acute viral respiratory tract infection, suggesting that even if exclusive breastfeeding is the recommended feeding method within the first 6 months, partial breastfeeding may also provide some protection against lower respiratory tract infections [40]. A Danish cohort study confirmed that the risk of hospitalization due to any infection in the first year of life decreased with a longer duration of any breastfeeding. Compared with never or partially breastfed group, exclusive breastfed infants for ≥4 months had a significant reduced risk (−55%) of hospital admissions for any infection for the first 24 to 36 months of life. Considering infection types, every extra month of any breastfeeding lowered the risk of lower respiratory tract and other infections (5% for both). On the contrary, no protective associations were found between breastfeeding and infection symptoms registered at home from ages 12 to 36 months [54]. Similarly, another cohort study found that protection against infections conferred by breastfeeding was limited to the first 12 months of life. Indeed, the higher risk of hospitalization was observed in breastfed children ≤ 6 months compared to ≥12 months (relative risk 1.22), but with similar risks for 6 to 11 months versus ≥12 months. Considering the time of weaning, breast-fed children who received complementary foods at 4 to 6 months of age had similar risk for infection as those receiving complementary foods after 6 months [51]. Conversely, a large cohort study (5322 children) highlighted that breastfeeding for ≥ 6 months was significantly associated with a reduced risk of lower respiratory tract infection (OR 0.71) up to 4 years of age [41], suggesting that breastfeeding effect against respiratory infections may persist beyond the first year of life.

Two other studies confirmed that breastfeeding significantly reduced hospitalization risk due to infections. A Japanese longitudinal study (43,367 children) showed that human milk was associated with reduced risk of hospitalization for respiratory infections (but not diarrhea) during second infancy (between 30 and 42 months of life) [44], and a cohort study in Honk Kong (8327 subjects) found that breastfeeding for >3 months was associated with a lower risk of hospital admission in the first 6 months of life for respiratory infections, gastrointestinal infections, or any infection [46].

Another large cohort study (15,809 infants from the UK Millennium Cohort Study) demonstrated that exclusive breastfeeding was associated with chest infections and diarrhea, but not with ear infections. Particularly, infants exclusively breastfed for <4 months had an increased risk of respiratory infection (risk ratios 1.24–1.28) and diarrhea (risk ratios 1.42–1.66) compared with the pre-2001 WHO policy (starting solids, but not formula, before 6 months, and continuing breastfeeding at 6 months). Moreover, this study found an excess risk of chest infections and diarrhea also among infants exclusively breastfed for 4–6 months, but who stopped breastfeeding by 6 months, highlighting the importance of continuing breastfeeding beyond 6 months of life [50].

Apparently contradictory results come from an Italian case–control study (496 infants aged < 6 months) reporting that exclusive breastfeeding at infant symptom onset was associated with a higher risk of viral respiratory infection (OR 3.7) confirmed by reverse transcriptase-polymerase chain reaction (RT-PCR). Breastfeeding may represent a proxy for closer contacts of the infant with the mother and, possibly, with other household members. However, in this study a longer breastfeeding period conferred a mild protection against viral respiratory infections (OR 0.98), suggesting that protective role of breastfeeding increases with duration [39]. This study also reinforced the importance of adopting Center for Disease Control and Prevention recommendations for the prevention of viral respiratory infections transmission to infants (symptomatic mothers should thoroughly wash their hands with soap and water before touching the infant and cover their nose and mouth with a tissue when sneezing or coughing in close contact with the infant) [76].

A protective effect of breastfeeding has been reported also against enterovirus infections responsible for hand, foot, and mouth disease. Interestingly, prolonged exclusive breastfeeding reduced the risk of developing fever, possibly due to some anti-inflammatory components of human milk that can reduce the production of pyrogenic substances. Moreover, breastfeeding can reduce infant discomfort conferring emotional support from the intimate contact with mother [31]. Finally, a Brazilian case–control study (267 infants < 6 months) confirmed that children exclusively breastfed and with mothers vaccinated against pertussis during pregnancy were 5 times less likely to develop a pertussis-like illness (OR 0.21) [35].

### 3.2. Breastfeeding and Gastrointestinal Infections

Some studies assessed the implication of natural breastfeeding on gastrointestinal infections in infancy (Table 1). Once again, human milk seems protective against diarrheal diseases development and severity as breastfeeding was significantly associated with reduced incidence/risk of acute gastroenteritis [42,43,45,47,49,50,52,55,57,67]. A meta-analysis of 18 studies found that not breastfeeding was associated with an increased risk of diarrhea mortality in comparison to exclusive breastfeeding among infants aged <5 months and to any breastfeeding among children aged 6–23 months (relative risk 10.52 and 2.18, respectively) [77]. This meta-analysis reinforced the importance of adopting WHO recommendation for exclusive breastfeeding during the first 6 months of life as a key child survival intervention, especially in developing countries.

Few studies evaluated the association between breastfeeding and hospitalization risk, with conflicting results. Tarrant, M. et al. found that breastfeeding for at least 3 months was associated with a lower risk of hospital admission in the first 6 months of life for gastrointestinal infections [46], while Davisse-Paturet, C. et al. showed that a shorter duration of breastfeeding (any breastfeeding for less than 3 months) was associated with higher risks of hospitalizations from gastrointestinal infections [53]. Differently, in a Japanese longitudinal study breastfeeding was not associated with reduced risk of hospitalization for diarrhea [44]. Interestingly, a Japanese cohort study (31,578 term and late-preterm infants; follow-up 18 months of life) found that exclusively breastfed late preterm infants did not show an increased risk of hospitalization for gastrointestinal infection, suggesting that exclusive breastfeeding probably mitigates the adverse effect of late preterm birth on gastrointestinal infections [48].

As for respiratory tract infections, breastfeeding duration seems to influence the risk of contracting gastrointestinal diseases. Raheem, R.A. et al. found that infants who are predominantly breastfed for longer duration have lower risks of having diarrhea [45]. In comparison with never breastfeeding, predominant breastfeeding for 4–6 months was associated with lower risk of gastroenteritis in the first 6 months of life (OR 0.34); this finding may at least in part be explained by exposure to higher doses of long-chain polyunsaturated fatty acids received from colostrum and human milk [49]. Finally, if considering specifically Rotavirus infection, a meta-analysis of six studies (3466 children) found that exclusive breastfeeding significantly reduces the risk of Rotavirus infection (OR 0.62) among children below 2 years of age [78].

### 3.3. Breastfeeding and Immunodeficiency Virus

Breastfeeding promotion for infants born from immunodeficiency virus (HIV)-infected mothers is a still highly debated topic, with important repercussions for public health strategies [79]. WHO in 2010 first recommended antiretroviral therapy (ART) to prevent HIV postnatal transmission through breastfeeding [80]. Subsequently, lifelong ART has been recommended for everyone from the time when any adult (including pregnant and breastfeeding women) or child is first diagnosed with HIV infection [81]. However, national guidelines for high-income countries generally discouraged women living with HIV from breastfeeding their infants. In 2013 the American Academy of Pediatrics recommended that pregnant women need to be aware of the potential risk of HIV transmission to infants from breastfeeding. In the United States, HIV-infected women should be counseled not to breastfeed, regardless of ART use or viral load. Moreover, HIV seronegative women who are at high risk of seroconversion should repeat HIV testing and receive education about the risk of HIV transmission through human milk and should be provided an individualized recommendation concerning the appropriateness of breastfeeding [82]. Similarly, the Centers for Disease Control and Prevention (CDC) recommended that HIV-infected mothers completely avoid breastfeeding their infants, regardless of ART and maternal viral load, providing feeding guidance and emotional support for mothers living with HIV that experienced social or cultural pressure to breastfeed. Indeed, CDC pointed out that keeping an undetectable viral load significantly reduces, but does not completely eliminate, the risk of transmitting HIV through breastfeeding [83].

On the contrary, in 2016 WHO recommended that HIV-infected mothers (and whose infants are HIV uninfected or of unknown HIV status) should exclusively breastfeed for the first six months of life. Mothers living with HIV should breastfeed for at least 12 months and may continue breastfeeding for up to 24 months or longer (similar to the general population) while being supported for ART adherence. WHO highlighted that this guideline is intended mainly for low- and middle-income countries with high HIV prevalence and settings in which diarrhea, pneumonia, and undernutrition are common causes of infant and child mortality [84]. Despite this clear division between recommendations for high- and low-income countries, breastfeeding from mothers living with HIV has been recommended also in high-resource settings [85,86]. Moreover, a recent systematic review reiterated that exclusive breastfeeding had a positive outcome on growth and development of all infants irrespective of HIV status [87]. Therefore, in absence of definitive and universally shared recommendations, health care professionals should provide adequate counseling and support to women living with HIV who desire to breastfeed, discussing benefit-risk ratio and supervising adherence to ART [88].

### 3.4. Breastfeeding during COVID-19 Pandemic

As well as many aspects of daily life and relationships, COVID-19 pandemic significantly affected women’s possibility to breastfeed and breastfeeding rate [89]. Despite the well-known positive effects of human milk for mother–child dyad, some concerns initially raised regarding the increased risk of vertical and early post-natal severe acute respiratory syndrome coronavirus 2 (SARS-CoV-2) transmission from positive women to newborns. However, breastfeeding is safe for infants and young children even when mothers are suspected or known to have COVID-19 [90].

Recent studies suggested that SARS-CoV-2-positive pregnant women do not expose their babies to an increased risk of infection via maternal nursing and breastfeeding. As COVID-19 is not effectively transmitted through breast milk [91], infected neonates might acquire the infection via the respiratory route due to maternal postnatal contact. For this reason, international organizations recommended to adopt strict proper hygienic and safety measures to minimize the risk of infant infection by droplets and direct contact with infected mother [92,93]. A prospective observational study showed that pregnant women with COVID-19 were at significant higher risk of gestational hypertension and preterm birth than general pregnant population, but their newborns did not have negative outcomes, except for prematurity. Particularly, only 5.4% of newborns were infected with SARS-CoV-2, no amniotic fluid samples were positive for SARS-CoV-2, and only 1.01% of PCR tests from breast milk were positive [94]. Therefore, separation of newborns from their mothers is not recommended since do not seem to be at increased risk of SARS-CoV-2 transmission. Conversely, keeping mothers and babies together is essential to promote breastfeeding [95]. A systematic review analyzed COVID-19 impact on breastfeeding, encouraging direct breastfeeding with appropriate protective measures. If mother–baby separation is unavoidable due to poor mother’s health condition, newborn should be fed with pumped breast milk or pasteurized donor breast milk until breastfeeding can be resumed [96]. Another recent systematic review showed increased positivity rates of SARS-CoV-2 among newborns who were breastfed and roomed-in (almost all remained asymptomatic), but there were no differences in SARS-CoV-2 positivity rates in neonates who received skin to skin care or delayed cord clamping [97]. A recent Italian study confirmed that vertical and perinatal SARS-CoV-2 infection is rare, and breastfeeding does not increase the risk of COVID-19 [98]. Considering that breastfed children acquired protection against various infections, also beyond the first year of life, it has been supposed that human milk may also decrease the risk of contract COVID-19 in infancy. About this, a Spanish observational enrolled 691 (age < 14 years) during a 5-month period (August–December 2020) founding a higher prevalence of positive SARS-CoV-2 RT-PCR test results among those who had been exclusively formula-fed compared with those who had been ever breastfed (OR 2.48) [99].

COVID-19 vaccination is compatible with breastfeeding, as clearly indicated by Infant Feeding in Emergencies Core Group, UNICEF, WHO [90], and by Italian scientific societies [100]. Lactating women should not be invited to stop breastfeeding to be vaccinated against COVID-19, and it is unlikely that vaccination has any impact on women’s ability to breastfeed. Despite this recommendation, a recent systematic review showed that vaccine hesitancy is still high in USA breastfeeding women [101]. To effectively counteract vaccine hesitancy, it is essential that health care providers provide evidence-based information and counseling to pregnant and lactating women [102].

A recent longitudinal study evaluated 64 lactating women with COVID-19 over a 2-month period beginning as early as the week of diagnosis. SARS-CoV-2 was not detected in breast milk, while most (75%) milk samples contained IgA specific to the SARS-CoV-2 spike glycoprotein receptor binding domain. These results supported recommendations encouraging lactating women to continue breastfeeding during and after COVID-19 illness [103]. Studies evaluating the presence of SARS-CoV-2-specific antibodies in breast milk after mRNA vaccination found that the majority of lactating mothers showed detectable neutralizing SARS-CoV-2 antibodies in breast milk, confirming possible passive immunization of breasted infants [104,105,106]. A longitudinal prospective study showed that human milk SARS-CoV-2-specific IgG levels peaked 1 month post-mRNA vaccination and persisted for at least 6 months, while SARS-CoV-2-specific IgA was detected up to 3 months but waned by 6 months. Neutralizing activity was seen in 83.3%, 70.4%, and 25.0% of milk samples at 1, 3, and 6 months post-vaccination. This study also showed that human milk samples showed similar IgG and neutralizing activity pre- and post-pasteurization, suggesting that also donor milk may confer passive immunization [107]. Recently, another prospective study compared IgA and IgG response and neutralizing activity against SARS-CoV-2 in human milk between lactating and vaccinated women out to 90 days after infection or vaccination. Interestingly, infection was associated with a highly variable IgA-dominant response while vaccination was associated with an IgG-dominant response; however, human milk exhibited neutralizing activity against SARS-CoV-2 virus in both cases [108].

## 4. Vitamin D Supplementation in Childhood

The term “vitamin D” is commonly used to indicate two different forms which are found in nature: vitamin D_3_ (cholecalciferol) from animal sources and vitamin D_2_ (ergocalciferol) from plants. Humans can produce vitamin D_3_ in their skin in response to sunlight exposure, while vitamin D_2_ and D_3_ may be obtained from dietary sources. Vitamin D is usually called the sunshine vitamin because most of the vitamin D we synthesize (90%) derives from skin exposure to solar ultraviolet B radiation, while the contribution of dietary intakes, with the exclusion of artificially fortified foods, may be considered negligible [109,110].

Vitamin D supplementation is the simplest and safest way to prevent nutritional rickets and, more generally, vitamin D deficiency at every age of life. Besides this historically well-known indication, considering the growing interest on skeletal and extra-skeletal actions of vitamin D, supplementation has been proposed to promote both bone and general health of children and adults, even if actual evidence from human studies suggest that supplementation of vitamin D-replete individuals does not provide demonstrable health benefits [111].

Vitamin D supplementation is essential to ensure an adequate vitamin D status during the first year of life, because newborns and infants should be poorly exposed to solar light and vitamin D content of breast and formula milk are both insufficient. Even if breast milk is the best food to satisfy children’s nutritional needs, it contains a poor amount of vitamin D (<50 IU/L) [112]. On the other hand, vitamin D intake of non-breastfed infants depends on vitamin D formula content (about 400 IU/L) and daily formula intake. Considering water requirements, formula-fed infants may receive 400 IU/day of vitamin D only when they weigh 5–6 Kg, so only some months after birth and near weaning, when daily milk consumption inevitably reduces [9]. Finally, as fetal vitamin D stores depend exclusively on maternal vitamin D status, newborns from mothers not receiving vitamin D supplementation and with poor sun exposure are at increased risk of vitamin D deficiency [113]. For all these reasons, first an expert position statement [9], followed by international [114] and national consensus [115] recommended vitamin D supplementation with 400 IU/day for all infants from birth to 12 months of life, independently of their mode of feeding. A recent meta-analysis (19 studies with 2837 mother–infant pairs) confirmed that vitamin D supplementation with 400 IU/day was effective to prevent vitamin D deficiency in high-risk term breastfed infants [116]. Another meta-analysis (28 trials with an overall sample size of 5908 participants of maternal–infant dyads) found that maternal postpartum or infant intermittent vitamin D supplementation may represent plausible substitutes for daily infant vitamin D supplementation in breastfed infants, but actual evidence remains too weak to support a policy update [117] and daily infant vitamin D supplementation remained mandatory during first and second infancy.

As nutritional rickets may develop during the entire pediatric age and an inadequate vitamin D status may negatively affect bone health, beyond 1 year of age vitamin D supplementation with at least 600 IU/day is recommended in children and adolescents with risk factors for deficiency [114]. A recent review confirmed that universal vitamin D supplementation until 12 months of age is strongly recommended, while beyond 1 year of life supplementation is recommended only in at-risk children. However, the authors highlighted that this age cut off is essentially arbitrary and not based on robust evidence, therefore the length of supplementation should always be individualized [118]. Risk factors for hypovitaminosis D identified from the Italian Pediatric Society are resumed in Table 2, while Table 3 summarizes the indications for vitamin D supplementation during childhood [115].

### 4.1. Vitamin D and Infections

Vitamin D, due to its complex immunoregulatory properties, modulates innate and adaptive immunity and inflammatory response. A detailed discussion of the immunological effects of vitamin D is beyond the scope of this review and can be found elsewhere [11,119,120]. Briefly, vitamin D stimulates innate immunity by increasing the production of cathelicidin and β-defensins, as well as enhancing chemotaxis and phagocytosis. At the same time, vitamin D reduces the synthesis of pro-inflammatory cytokines (IL-1, IL-6, TNF-α) and Th1 and Th17 cells response, favoring Th2 cells activity with consequent anti-inflammatory effect due to increased production of IL-4, IL-5, IL-10, IL-13 [119].

Several observational studies found a relationship between vitamin D status and incidence or severity of upper- and lower-respiratory tract infections in children, both in developing and in westernized countries [121,122,123]. However, a possible association between severe vitamin D deficiency and respiratory tract infections was historically hypothesized due to the identification of a significant increased risk of pneumonia and respiratory complications in rachitic children, a condition known as rachitic lung [124,125,126]. A significant association between 25-hydroxyvitamin D [25(OH)D] levels and other pediatric infections has also been found, including urinary tract infections [127], otitis media [128], acute diarrhea [129], rotavirus infection [130], malaria [131], leishmaniosis [132], hepatitis C [133], and sepsis [134,135,136]. Moreover, some studies investigated a possible relationship between vitamin D deficiency and tuberculosis infection in children, with discordant results [137,138]. Even if vitamin D supplementation does not seem to have any beneficial effect in the treatment of tuberculosis in children and adults [139], an individual-participant data meta-analysis showed that vitamin D predicts tuberculosis disease risk in a dose-dependent manner and tuberculosis risk was highest among HIV-positive individuals with severe vitamin D deficiency [140].

Despite a growing number of studies assessing the relationship between vitamin D status and infections risk, it is still unclear whether vitamin D deficiency should be considered a consequence of the infection or if it plays a causative role in increasing infections risk. More robust evidence was expected from supplementation studies, but several variables may confound the results and complicate the comparison between different studies (i.e., differences in population enrolled, vitamin D supplementation dosage and regimen, length of follow-up, and percentage of enrolled individuals with severe vitamin D deficiency).

In Table 4 we reported the meta-analyses that evaluated the association between vitamin D status or vitamin D supplementation with infections risk or severity in children [127,128,134,135,141,142,143,144,145,146,147,148,149,150,151,152,153,154,155,156]. Most of meta-analyses confirmed a significant protective role of vitamin D supplementation against respiratory infections; particularly, major benefits were observed in children and adolescents, asthmatic subjects, individuals with severe vitamin D deficiency, and those receiving a daily dosing regimen (400–1000 IU/day) for a duration of 12 months or less.

A systematic review of observational studies and randomized controlled trials (RCTs) focusing on extra-skeletal actions of vitamin D confirmed that vitamin D supplementation plays a significant role in the primary prevention of acute respiratory infections [157]. Preventive efficacy of vitamin D supplementation was particularly evident in subjects with severe deficiency [25(OH)D < 10 ng/mL], while vitamin D administration was not effective as adjunctive treatment of acute respiratory infections [155,156,157]. An expert consensus statement from the World Association of Infectious Diseases and Immunological Disorders confirmed that vitamin D could play a role in children with recurrent respiratory infections. However, future large and methodologically adequate studies in predisposed children are needed to clearly identify the lowest serum vitamin D level associated with a significant increased risk of respiratory infections, in adjunct with the most effective dosage, regimen and duration of vitamin D supplementation [158]. Similarly, an Italian inter-society consensus on the prevention of recurrent respiratory infections found that reduced vitamin D levels are associated with an increased incidence of viral respiratory infections in the first years of life [159]. Even if the evidence was too low to universally recommend vitamin D supplementation only for the prevention of respiratory infections, populations with low socioeconomic status and severe vitamin D deficiency, and children with recurrent acute otitis may benefit from vitamin D supplementation for such purpose. Finally, a recently published review of meta-analyses and RCTs confirmed that individuals most likely to benefit from supplementation are those with baseline vitamin D deficiency or with selected high-risk conditions [160].

Even if vitamin D supplementation is universally recommended during the first year of life, all studies included in Table 1 except one [41] did not assess the adherence with vitamin D supplementation in enrolled children. Similarly, no meta-analyses on vitamin D and infections risk (Table 4) considered the type of feeding of enrolled pediatric patients and did not distinguish between breastfed and formula-fed infants. Thus, all these studies cannot provide any information regarding a possible positive synergic effect of vitamin D supplementation and breastfeeding on infections prevention during childhood.

### 4.2. Vitamin D and COVID-19

Severe forms of COVID-19 are characterized by an increase in IL-1, IL-6, and TNF-α (the so-called cytokine storm) with consequent acute respiratory distress syndrome. An adequate vitamin D status could be protective both increasing innate immune response (with a reduction in the risk of contracting SARS-CoV-2) and exerting anti-inflammatory effects [161]. Indeed, some authors suggested that vitamin D status may be considered a prognostic indicator for morbidity and mortality in patients with COVID-19 [162]. However, the link between vitamin D deficiency and severe COVID-19 may represent only an association rather than being causative because these two conditions share some risk factors such as overweight-obesity, old age, and belonging to ethnic minorities [163].

SARS-CoV-2 pandemic has been proposed as a new risk factor for vitamin D deficiency in childhood [136,164], as the measures adopted to contain the spread of COVID-19 (e.g., lockdown, repeated quarantine periods, school closure, distance education) significantly increased screen time and reduced sun exposure of children and adolescents [165]. A Korean pediatric study (age 4–14 years) confirmed the association between vitamin D, obesity, and COVID-19. The comparison before and after pandemic onset demonstrated an increase in both the prevalence of overweight-obesity (from 23.9% to 31.4%) and a significant reduction in circulating levels of 25(OH)D [166].

Children with COVID-19 had significantly lower serum vitamin D levels than age-matched controls [167], and vitamin D deficiency was the most common vitamin deficiency in affected individuals [25(OH)D < 20 ng/mL in 82% of patients aged 1 month to 18 years] [168]. A significant association between vitamin D deficiency, clinical severity, and inflammation markers was found also in pediatric COVID-19 cases [169]. Furthermore, it has been suggested that vitamin D deficiency could increase the risk of developing multisystem inflammatory syndrome in children (MIS-C), a rare severe complication of childhood COVID-19 [170]. At present, one UK study evaluated serum 25(OH)D levels in children with MIS-C reporting that most patients were of Black, Asian, and Minority Ethnic origin (16/18; 89%) and had severe vitamin D deficiency [13/18 (72%) with 25(OH)D levels < 12 ng/mL]. Moreover, mean 25(OH)D levels of the whole cohort were significantly lower when compared with the 2014/2015–2015/2016 National Diet and Nutrition Survey mean 25(OH)D for children [171]. Considering the high prevalence of vitamin D deficiency in this small MIS-C cohort, the authors recommended continuous vitamin D supplementation of high-risk children and adolescents.

A recent systematic review (including 6 studies and 2 reviews for a total of 271 children and adolescents) found that 46% of pediatric subjects with COVID-19 had vitamin D deficiency. Moreover, vitamin D deficiency significantly increased the risk of developing severe COVID-19 by 5.5 times. However, these results derive from the analysis of only 102 cases, so larger studies are needed. The absent definition of the serum 25(OH)D cutoff level used to identify vitamin D deficiency is another limitation of this systematic review [172]. Previously, we discussed that breastfeeding may contribute to decrease the risk of contract COVID-19 in infancy. Interestingly, it has been demonstrated that vitamin D concentration in human milk was higher in women without infection than in women with viral symptoms or with confirmed COVID-19, suggesting that vitamin D level in breast milk may influence maternal immunity against COVID-19 infection [173].

On December 2020, the National Institute for Health and Care Excellence (NICE) published a guideline on vitamin D and COVID-19, recommending that all adults (including pregnant or breastfeeding women), young people, and children over 4 years should consider vitamin D supplementation at 400 UI/day at least during winter (between October and early March). Supplementation should be offered throughout the year to people with insufficient sunshine exposure (also as consequence of restriction due to COVID-19 pandemic) or with dark skin [174]. After reviewing available evidence, NICE recommended to not prescribe vitamin D supplementation solely to prevent or treat COVID-19, except as part of a clinical trial.

## 5. Conclusions

The worldwide promotion of natural breastfeeding can be considered the simplest, cheapest, safest, and most ecological primary prevention strategy to reduce the risk of contracting infectious diseases during childhood. The benefits of breastfeeding know no barriers, as demonstrated by the positive results of studies conducted in different continents and in various ethnic groups. Exclusivity and duration of breastfeeding represent the main determinants of the protective effect of human milk, starting from the first days of life, independently from gestational age, delivery mode, and birth weight. Therefore, every child has the right to benefit from the several advantages conferred by breastfeeding, obviously including the reduction of getting sick from an infectious disease. Human milk can protect against a large multiplicity of infections, particularly those involving respiratory and gastrointestinal tract. Even if most of the studies found that breastfeeding protection is limited to the first 6–12 months of life, some authors reported that some protective effect last also during second and third infancy. The effort to protect and promote breastfeeding must be increased during SARS-CoV-2 pandemic, for example to counteract the recent increase in cases of bronchiolitis (taking advantages from the protective effect of human milk against RSV infection), and more generally to safeguard the inviolable relationship between mother and child jeopardized by COVID-19.

Recent studies suggested a significant association between vitamin D status and severity or incidence of various childhood infectious diseases, particularly respiratory infections. However, despite growing evidence resulted from meta-analyses of supplementation studies, at present vitamin D supplementation should not be universally recommended exclusively to prevent infectious diseases, including COVID-19. Therefore, we recommend against routine 25(OH)D testing in children with infectious diseases, including recurrent respiratory infections. On the other hand, risk factors for vitamin D deficiency should be carefully and periodically monitored by pediatricians, and vitamin D supplementation should be offered to all at-risk children, particularly during winter, to prevent severe vitamin D deficiency [175]. A long-lasting unrecognized vitamin D deficiency may negatively affect bone health and, at the same time, represent a significant risk factor for developing severe infectious diseases, including COVID-19 and MIS-C. Daily vitamin D supplementation at doses recommended for age (400 IU/day from birth to 12 months and 600 IU/day from 1 to 18 years) seems to offer better results in terms of protection against infections than intermittent or high-dose vitamin D administration.

Unfortunately, published studies did not evaluate the possible positive synergistic effect of exclusive breastfeeding and vitamin D supplementation on prevention of common childhood infections. Future research should focus on this topic, to definitively address the importance of promoting these two simple public health strategies.

*Authors’ Opinions*

- Breastfeeding promotion, protection, and support should be considered fundamental public health objectives.
- Every child has the right to benefit from breastfeeding advantages, including a significant reduction in infection risk during childhood.
- Breastfeeding duration and exclusivity represent the main determinants of human milk protective role against infections.
- Vitamin D deficiency may be considered a modifiable risk factor for developing infectious diseases during childhood.
- Vitamin D supplementation is the simpler and more effective strategy to prevent vitamin D deficiency.
- Vitamin D supplementation with at least 400 IU/day should be proposed to every infant in the first year of life. Older children should receive supplementation with at least 600 IU/day in presence of risk factors for vitamin D deficiency.

## Figures and Tables

**Table 1:** Studies that evaluated the association between breastfeeding and infection risk in childhood.

Author, Year of Publication	Type of Infection	Country/Continent	Type of Study	Cases, n	Length of Follow-Up/Age of Enrolled Children/Data of Literature Search	Results
Zhu, Q. et al., 2012 [31]	Hand, foot, and mouth disease	China	Cross-sectional	372	Age of children: 6 months–6 years	Prolonged exclusive breastfeeding (OR 0.401) was a protective factor for the incidence of fever.
Netzer-Tomkins, H. et al., 2016 [32]	Neonatal fever	Israel	Retrospective case–control	140	Age of infant: <1 month	Hospitalized children had a 2.5-fold increased risk of not being exclusively or predominantly breastfed (OR 2.49).
Boccolini, C.S. et al., 2011 [33]	Pneumonia	Brazil	Ecological	642,792	Age of infants: <1 year	Breastfeeding prevalence among children between 9 and 12 months old and exclusive breastfeeding prevalence among children under 6 months old were associated with a lower rate ratio of hospitalization for pneumonia (rate ratio 0.62 and 0.52, respectively).
Nascimento, R.M.D. et al., 2021 [34]	Pertussis-like illness	Brazil	Case–control	267	Age of infants: <6 months	The protective effect of breastfeeding was of 74%. Children younger than six months, who were exclusively breastfed and with mothers vaccinated against pertussis during pregnancy were 5 times less likely to develop pertussis-like illness, corresponding to a protection of 79%.
Jang, M.J. et al., 2020 [35]	RSV bronchiolitis	Korea	Retrospective study	411	Age of infants: <1 year	The OR for oxygen therapy was significantly higher in the artificial-milk-formula-fed group than in the breast milk feeding group (adjusted OR 3.807).
Gómez-Acebo, I. et al., 2021 [36]	Bronchiolitis	Spain	Cohort	969	Length of follow-up: 1 year of life	At 4 months, exclusive breastfeeding reduced the number of episodes of bronchiolitis by 41% (IR 0.59) and mixed feeding by 37% (IR 0.63). An early swap to mixed breastfeeding before months 2 or 4 was associated with a reduced number of episodes of bronchiolitis when compared with infant formula alone.
Jansen, S. et al., 2020 [37]	Respiratory infections	Indonesia	Retrospective case–control	100	Age of infants: 7–12 months	Non-breastfed infants were at 14 times greater risk of contracting respiratory infections.
Wang, J. et al., 2017 [38]	Respiratory infections	UK	Cohort	4040	Length of follow-up: 2 years of life	Breastfeeding for >6 months was protective against bronchiolitis (OR 0.72).
Pandolfi, E. et al., 2019 [39]	Respiratory infections	Italy	Case–control	496	Age of infants: <6 months	Exclusive breastfeeding at symptom onset was associated with a higher risk of viral respiratory infection in the first 6 months of life (OR 3.7), but protection increased with breastfeeding duration (OR 0.98).
Vereen, S. et al., 2014 [40]	Respiratory infections	USA	Cross-sectional	629	Median infant age: 3 months	Breastfeeding (ever vs. never) was associated with decreased relative odds of a lower versus upper acute viral respiratory tract infection in the first year of life (OR 0.64).
Tromp, I. et al., 2017 [41]	Respiratory infections	The Netherlands	Cohort	5322	Length of follow-up: 4 years of life	Breastfeeding for ≥6 months was significantly associated with a reduced risk of lower respiratory tract infection up to 4 years of age (OR 0.71).
Zivich, P. et al., 2018 [42]	Respiratory infections and diarrhea	Democratic Republic of Congo	RCT	931	Length of follow-up: 6 months of life	Implementation of Baby-Friendly Hospital Initiative steps 1–9 was associated with a decreased incidence of reported diarrhea (IRR 0.72) and respiratory illness (IRR 0.48), health facility visits due to diarrhea (IRR 0.60) and respiratory illness (IRR 0.47) in the first 6 months of life.
Guo, C. et al., 2020 [43]	Respiratory infections and diarrhea	China	Longitudinal	41	Length of follow-up: 1 years of life	Breastfeeding was significantly associated with a lower incidence of respiratory infections and diarrhea in children born from vaginal delivery or cesarean section.
Yamakawa, M. et al., 2015 [44]	Respiratory infections and diarrhea	Japan	Longitudinal	43,367	Length of follow-up: 42 months of life	Breastfeeding was not associated with reduced risk of hospitalization for diarrhea. Breastfeeding was associated with reduced risk of hospitalization for respiratory infections between ages 30 and 42 months (OR of exclusive breastfeeding 0.76).
Raheem, R.A. et al., 2017 [45]	Respiratory infections and diarrhea	Australia	Cohort	458	Length of follow-up: 6 months of life	The risk of acquiring respiratory infections is significantly reduced when the infants were predominantly breastfed for 3 months (OR 0.56) and 6 months (OR 0.45). The risk of getting diarrhea is significantly reduced even when the babies were partially breastfed for 6 months (OR 0.31).
Tarrant, M. et al., 2010 [46]	Respiratory and gastrointestinal infections	Honk Kong	Cohort	8327	Length of follow-up: 8 years of life	Breastfeeding for ≥3 months was associated with a lower risk of hospital admission in the first 6 months of life for respiratory infections (hazard ratio 0.64), gastrointestinal infections (0.51), and any infection (0.61).
Frank, N.M. et al., 2019 [47]	Respiratory and gastrointestinal infections	USA	Prospective longitudinal study	6861	Length of follow-up: 4 years of life	At 3–6 months of age, breastfeeding was found to be inversely associated with the odds of respiratory infections with fever (OR 0.82), otitis media (OR 0.76), and infective gastroenteritis (OR 0.55). Between 6 and 18 months of age, breastfeeding continued to be inversely associated with the odds of ear infection and infective gastroenteritis, and additionally with the odds of conjunctivitis, and laryngitis and tracheitis.
Nakamura, K. et al., 2020 [48]	Gastrointestinal infections	Japan	Cohort	31,578	Length of follow-up: 18 months of life	Exclusively breastfed late preterm infants did not show an increased risk of hospitalization for gastrointestinal infection.
Morales, E. et al., 2012 [49]	Various infections	Spain	Cohort	580	Length of follow-up: 14 months of life	In comparison with never breastfeeding, predominant breastfeeding for 4–6 months was associated with lower risk of wheezing (OR 0.53), low respiratory tract infections (OR 0.51) and atopic eczema (OR 0.58) between months 7 and 14 of life. Predominant breastfeeding for 4–6 months was associated with lower risk of gastroenteritis during the first 6 months of life (OR 0.34)
Quigley, M.A. et al., 2016 [50]	Various infections	UK	Cohort	15,809	Length of follow-up: 9 months of life	Exclusive breastfeeding for <4 months was associated with an increased risk of chest infection (risk ratios 1.24–1.28) and diarrhea (risk ratios 1.42–1.66). There was also an excess risk of the chest infection (risk ratios 1.19) and diarrhea (risk ratios 1.66) among infants exclusive breastfed for 4–6 months who stopped breastfeeding by 6 months.
Størdal, K. et al., 2017 [51]	Various infections	Norway	Cohort	70,511	Length of follow-up: 18 months of life	Higher risk of hospitalization was observed in breastfed children ≤ 6 months compared to ≥12 months (RR 1.22).
Davisse-Paturet, C. et al., 2020 [52]	Various infections	France	Cohort	1603	Length of follow-up: 8 years of life	Compared with never breastfed infants, ever-breastfed infants were at a lower risk of diarrhea events in early infancy as well as infrequent events of bronchitis/bronchiolitis throughout infancy. Only predominant breastfeeding duration was related to frequent events of bronchitis/bronchiolitis and infrequent events of otitis.
Davisse-Paturet, C. et al., 2019 [53]	Various infections	France	Cohort	9703	Length of follow-up: 2 years of life	Any breastfeeding for <3 months was associated with higher risks of hospitalizations from gastrointestinal infections or fever. Predominant breastfeeding for <1 month was associated with higher risk of a single hospital admission. Ever breastfeeding was associated with lower risk of antibiotic use.
Christensen, N. et al., 2020 [54]	Various infections	Denmark	Cohort	815	Length of follow-up: 3 years of life	Adjusted incidence rate ratio (IRR) for hospitalization due to any infection decreased with a longer duration of any breastfeeding (IRR 0.96; 0.88 for exclusively breastfed infants). The strongest associations between the duration of any breastfeeding and hospitalizations due to infection were found within the first year of life and for lower respiratory tract infections.
Ardiç, C. et al., 2018 [55]	Various infections	Turkey	Cohort	411	Length of follow-up: 5 years of life	Infants breastfed longer than 12 months had less acute otitis media and acute gastroenteritis when compared with the infants breastfed less than 12 months.
Li, R. et al., 2014 [56]	Various infections	USA	Prospective longitudinal	1281	Length of follow-up: 6 years of life	Children breastfed for ≥9 months had lower odds of past-year ear (OR 0.69), throat (OR 0.68), and sinus (OR 0.47) infections compared with those breastfed >0 to <3 months.
Mulatu, T. et al., 2021 [57]	Various infections	Ethiopia	Nationally representative survey	1034	Age of infants: <6 months	Compared to infants who were non-exclusively breastfed, the odds of having an illness with fever in the last 2 weeks among infants who were exclusively breastfed decreased by 66% (OR 0.34). Exclusively breastfed infants had lower odds of having an illness with cough (OR 0.38) and having diarrhea (OR 0.33) compared to non-exclusively breastfed infants.
Ladomenou, F. et al., 2010 [58]	Various infections	Greece	Prospective study	926	Length of follow-up: 12 months of life	Infants exclusively breastfed for 6 months presented with fewer infectious episodes than their partially breastfed or non-breastfed peers (OR 0.58 for respiratory infections and 0.37 for acute otitis media). Prolonged exclusive breastfeeding was associated with fewer infectious and fewer admissions to hospital for infection in the first year of life.

OR: odds ratio; RR: relative risk; IRR: incidence rate ratio; IR: incidence ratio; RCT: randomized controlled trial.

**Table 2:** Risk factors for vitamin D deficiency in childhood [115].

First Year of Life	1–18 Years
Non-Caucasian ethnicity with dark skin pigmentation	
Inadequate diets (i.e., vegan diet)	
Chronic kidney disease	
Hepatic failure and/or cholestasis	
Malabsorption syndromes (i.e., cystic fibrosis, inflammatory bowel diseases, celiac disease at diagnosis)	
Chronic therapies: anticonvulsants, systemic glucocorticoids, antiretroviral therapy, systemic antifungals (i.e., ketoconazole)	
Infants born from mothers with multiple risk factors for vitamin D deficiency, particularly in absence of vitamin D supplementation during pregnancy	Reduced sunlight exposure (due to lifestyle factors, chronic illness or hospitalization, complex disability, institutionalization, covering clothing for religious or cultural reasons) and/or constant use of sunscreens
	International adoption
	Obesity

**Table 3:** Key points of vitamin D supplementation in childhood [115].

First Year of Life	1–18 Years
Vitamin D supplementation is recommended in all newborns, independently of the type of feeding.	Vitamin D supplementation is recommended in children and adolescents with risk factors for vitamin D deficiency.
Vitamin D supplementation should be started within the first days of life and continued throughout the first year.	Vitamin D supplementation is recommended from the end of fall to the beginning of spring (November–April) in children and adolescents with reduced sun exposure during summer. Continuous vitamin D supplementation is recommended in cases of permanent risk factors for vitamin D deficiency.
Infants born at term without risk factors for vitamin D deficiency should receive 400 IU/day of vitamin D. In the presence of risk factors for vitamin D deficiency up to 1000 IU/day of vitamin D can be given.	At-risk children should receive daily vitamin D supplementation ranging from 600 IU/day (i.e., in presence of reduced sun exposure) up to 1000 IU/day (i.e., in presence of multiple risk factors for vitamin D deficiency).
Daily administration of vitamin D is recommended.	In cases of poor compliance, supplementation with intermittent dosing (weekly or monthly doses for a cumulative monthly dose of 18,000–30,000 IU of vitamin D) can be considered, starting from children aged 5–6 years and particularly during adolescence.
Individuals on anticonvulsants, oral corticosteroids, antimycotics and antiretroviral drugs should receive at least 2–3 times more vitamin D than the daily requirement recommended for age.	
Vitamin D metabolites and their analogs (calcifediol, alfacalcidol, calcitriol, and dihydrotachysterol) are not recommended for the routine vitamin D supplementation.	
25(OH)D testing in children and adolescents is not recommended. Evaluation of serum 25(OH)D levels can be considered in presence of multiple risk factors for vitamin D deficiency. Vitamin D status should be monitored at least yearly in subjects that require continuous supplementation.	

**Table 4:** Meta-analyses that evaluated the association between vitamin D and infections in childhood.

Author, Year of Publication	Type of Infection	Type of Included Studies	Number of Included Studies	Patients (Cases + Controls), n	Results
Deng, Q.F. et al., 2019 [127]	Urinary tract infections	Association with vitamin D levels	9 studies (6 case–control, 2 cross-sectional, 1 RCT)	1921 (children and adults)	Vitamin D insufficiency was associated with a significantly increased risk of having a urinary tract infection (OR 3.01). Vitamin D level was significantly lower in the urinary tract infection group.
Li, X. et al., 2021 [141]	Urinary tract infections	Association with vitamin D levels	6 studies (1 cohort, 2 case–control, 2 prospective, 1 cross-sectional)	645 (children)	Serum vitamin D levels in children with urinary tract infections were significantly lower than healthy control children.
Li, H.B. et al., 2016 [128]	Otitis media	Association with vitamin D levels	5 studies (1 cohort, 2 case–control, 1 observational, 1 RCT)	16,689 (children and adults)	Participants with otitis media had lower level of plasma vitamin D when compared with controls. Serum vitamin D level was not associated with the risk of otitis media.
Xiao, D. et al., 2020 [134]	Sepsis	Association with vitamin D levels	13 studies (case–control, cohort, cross-sectional)	1745 (children)	The association between vitamin D deficiency and sepsis was significant (OR 1.13).
He, M. et al., 2021 [135]	Sepsis	Association with vitamin D levels	16 studies (observational)	2382 (children)	Vitamin D deficient patients had significantly higher sepsis (OR 2.35), pediatric risk of mortality III score (OR 2.19), higher length of hospital stay (OR 4.26) higher duration of mechanical ventilation (OR 1.89) compared with non-vitamin D deficient children.
Yu, W. et al., 2021 [142]	Sepsis	Association with vitamin D levels	27 studies (10 cohort, 17 case–control)	3314 in case–control studies (children)	In case–control studies, maternal and neonatal 25(OH)D level in sepsis group was significantly lower than non-sepsis group. The percentage of severe vitamin D deficiency was significant higher in sepsis group comparing to non-sepsis group (OR 2.66). In cohort studies, the incidence of sepsis in lower 25(OH)D group was 30.4% comparing with 18.2% in higher 25(OH)D level group.
Cariolou, M. et al., 2019 [143]	Sepsis and respiratory infections	Association with vitamin D levels	52 studies (cross-sectional, case–control, cohort)	7434 (children)	Mortality of 18 cohort studies (2463 total individuals) showed increased risk of death in 25(OH)D deficient children (OR 1.81). There were insufficient studies to meta-analyze sepsis and respiratory tract-related mortality.
Yakoob, M.Y. et al. 2016 [144]	Various infections	Vitamin D supplementation for prevention	4 studies (RCTs)	3198 (children)	Vitamin D supplementation did not influence death, the occurrence of the first or only episode of pneumonia, or on children with pneumonia. There was no obvious difference in the first or repeat episodes of diarrhea between supplemented and unsupplemented children.
Jat, K.R. et al., 2017 [145]	Lower respiratory infections	Association with vitamin D levels	12 studies (3 cohort, 2 cross-sectional, 7 case–control)	2279 (children)	Vitamin D deficiency was more prevalent in cases compared to controls (OR 3.29) and mean vitamin D levels in children with lower respiratory infections were significantly lower as compared to controls (−3.5 ng/mL).
Zhou, Y.F. et al., 2019 [146]	Pneumonia	Association with vitamin D levels	8 studies (4 case–control, 2 retrospective, 1 cross-sectional, 1 prospective)	20,966 (children and adults)	Patients with vitamin D deficiency [25(OH)D levels < 20 ng/mL)] had a significantly increased risk of pneumonia (OR 1.64) and a decrease of −5.63 ng/mL in serum vitamin D was observed in subjects with pneumonia.
Charan, J. et al., 2012 [147]	Respiratory infections	Vitamin D supplementation for prevention	5 studies (RCTs)	1868 (children and adults)	Events of respiratory tract infections were significantly lower in vitamin D group as compared to control group (OR 0.582). Vitamin D supplementation decreases the events related to respiratory tract infections. On separate analysis of clinical trials dealing with groups of children and adults, beneficial effect of vitamin D was observed in both (OR 0.579 and 0.653, respectively).
Bergman, P. et al., 2013 [148]	Respiratory infections	Vitamin D supplementation for prevention	11 studies (RCTs)	5660 (children and adults)	Vitamin D showed a protective effect against respiratory infections (OR 0.64). The protective effect was larger in studies using once-daily dosing compared to bolus doses (OR 0.51 vs. OR 0.86).
Mao, S. et al., 2013 [149]	Respiratory infections	Vitamin D supplementation for prevention	7 studies (RCTs)	4827 (children and adults)	The study does not support the routine use of vitamin D supplementation for respiratory infections prevention in healthy populations.
Xiao, L. et al., 2015 [150]	Respiratory infections	Vitamin D supplementation for prevention	7 studies (RCTs)	6503 (children)	The study indicates a lack of evidence supporting the routine use of vitamin D supplementation for the prevention of acute respiratory infections in healthy children. Supplementation may benefit children previously diagnosed with asthma (vitamin D supplementation resulted in a 74% reduction in the risk of asthma exacerbation, RR 0.26).
Vuichard Gysin, D. et al., 2016 [151]	Respiratory infections	Vitamin D supplementation for prevention	15 studies (RCTs)	7053 (children and adults)	In previously healthy individuals vitamin D supplementation does not reduce the risk of respiratory infections.
Martineau, A.R. et al., 2017 [152]	Respiratory infections	Vitamin D supplementation for prevention	25 studies (RCTs)	11,321 (children and adults)	Vitamin D supplementation reduced the risk of acute respiratory tract infection among all participants (OR 0.88). In subgroup analysis, protective effects were seen in those receiving daily or weekly vitamin D without additional bolus doses (OR 0.81). Among those receiving daily or weekly vitamin D, protective effects were stronger in those with baseline 25(OH)D levels < 10 ng/mL (OR 0.30).
Vlieg-Boerstra, B. et al., 2021 [153]	Viral respiratory infections	Vitamin D supplementation for prevention	19 studies (RCTs)	10,837 (children and adults)	Meta-analysis of the 6 studies in children showed a non-significant decreased incidence of respiratory infections with vitamin D supplementation. Meta-analysis of the seven studies amongst adults showed a significant decreased incidence of respiratory infections (Risk Ratio 0.89).
Jolliffe, D.A. et al., 2021 [154]	Respiratory infections	Vitamin D supplementation for prevention	43 studies (RCTs)	48,488 (children and adults)	A significantly lower proportion of participants in the vitamin D supplementation group had one or more acute respiratory infection (61.3%) than in the placebo group (62.3%; OR 0.92). Protective effects of supplementation were observed in trials in which vitamin D was given in a daily dosing regimen (OR 0.78), at daily dose equivalents of 400–1000 IU (OR 0.70), for a duration of 12 months or less (OR 0.82), and to participants aged 1.00–15.99 (OR 0.71). No significant effect of vitamin D supplementation was observed depending on baseline 25(OH)D levels.
Das, R.R. et al., 2018 [155]	Pneumonia	Vitamin D supplementation for treatment	7 studies (RCTs)	1529 (children)	The effects of vitamin D on outcomes were inconclusive when compared with control: time to resolution of acute illness, mortality rate, duration of hospitalization, and time to resolution of fever.
Yang, C. et al., 2021 [156]	Pneumonia	Vitamin D supplementation for prevention and treatment	13 studies (RCTs)	4786 (children and adults)	Vitamin D supplementation significantly reduced incidence of repeated episodes of pneumonia (Risk Ratio 0.68). Supplementation had more reducing effects on repeat episodes of pneumonia in trials in which the population were children (Risk Ratio 0.66), duration < 3 months (Risk Ratio 0.55), or dose < 300,000 IU (Risk Ratio 0.51). There was no statistical difference on recovery rate.

OR: odds ratio; RR: relative risk; RCT: randomized controlled trial.

## Abbreviations

25(OH)D	25-hydroxyvitamin D
ART	Antiretroviral therapy
BFHI	Baby Friendly Hospital Initiative
CDC	Centers for Disease Control and Prevention
COVID-19	Coronavirus disease 2019
HIV	Immunodeficiency virus
Ig	Immunoglobulin
MIS-C	Multisystem inflammatory syndrome in children
NICE	National Institute for Health and Care Excellence
OR	Odds ratio
RCT	Randomized controlled trial
RSV	Respiratory syncytial virus
RT-PCR	Reverse transcriptase-polymerase chain reaction
